# Transcriptomic Analysis Reveals Adaptive Responses of an Enterobacteriaceae Strain LSJC7 to Arsenic Exposure

**DOI:** 10.3389/fmicb.2016.00636

**Published:** 2016-05-02

**Authors:** Yingjiao Zhang, Songcan Chen, Xiuli Hao, Jian-Qiang Su, Ximei Xue, Yu Yan, Yong-Guan Zhu, Jun Ye

**Affiliations:** ^1^Key Lab of Urban Environment and Health, Institute of Urban Environment, Chinese Academy of SciencesXiamen, China; ^2^State Key Lab of Urban and Regional Ecology, Research Center for Eco-Environmental Sciences, Chinese Academy of SciencesBeijing, China; ^3^University of Chinese Academy of SciencesBeijing, China

**Keywords:** transcriptome, arsenic, RNA-seq, reactive oxygen species, nitric oxide, Enterobacteriaceae

## Abstract

Arsenic (As) resistance determinant *ars* operon is present in many bacteria and has been demonstrated to enhance As(V) resistance of bacteria. However, whole molecular mechanism adaptations of bacteria in response to As(V) stress remain largely unknown. In this study, transcriptional profiles of Enterobacteriaceae strain LSJC7 responding to As(V) stress were analyzed using RNA-seq and qRT-PCR. As expected, genes involved in As(V) uptake were down-regulated, those involved in As(V) reduction and As(III) eﬄux were up-regulated, which avoided cellular As accumulation. Reactive oxygen species and nitric oxide (NO) were induced, which caused cellular damages including DNA, protein, and Fe–S cluster damage in LSJC7. The expression of specific genes encoding transcriptional regulators, such as *nsrR* and *soxRS* were also induced. NsrR and SoxRS modulated many critical metabolic activities in As(V) stressed LSJC7 cells, including reactive species scavenging and repairing damaged DNA, proteins, and Fe–S clusters. Therefore, besides As uptake, reduction, and eﬄux; oxidative stress defense and damage repair were the main cellular adaptive responses of LSJC7 to As(V) stress.

## Introduction

Arsenic (As) is considered to be a ubiquitous toxic substance and carcinogen, released from both anthropogenic and natural sources, and has high environmental impact (Zhu et al., 2014). It ranks first on the US Priority List of Hazardous Substances by the US Environmental Protection Agency and Agency for Toxic Substances and Disease Registry^1^. Typical redox states of As in living organisms are pentavalent As(V) and trivalent As(III) (Cullen and Reimer, 1989). As a consequence of their pervasiveness, microorganisms have developed special adaptive, resistance, and colonization mechanisms to cope with the presence of As. Microorganisms usually avoid As toxicity through uptake selectivity, eﬄux of As, As(III) oxidation, As(V) reduction, and methylation and volatilization (Zhu et al., 2014). Typically, most bacteria can reduce As(V) to the more toxic and mobile As(III) then extrude, which is the most common As resistance mechanism depending on the presence of *ars* genes (Lin et al., 2006).

It has been reported that metal and metalloid ions can participate in redox reactions to induce toxicity (Harrison et al., 2009). The redox reactions induced by As stress can be divided into two types (Harrison et al., 2009): As(V) reduction–oxidation reaction with cellular thiols (RSH), and production of reactive oxygen species (ROS) via intermediate S radical chemistry (Lemire et al., 2013). The production of ROS, such as superoxide anion ($O_{2}^{–}$), hydrogen peroxide (H_2_O_2_), and hydroxyl radical (HO^∙^), serves as critical signaling events in cell proliferation and survival (Ray et al., 2012), and can also modify organic molecules to induce cytotoxicity. The dual functions of ROS play an important role in cells by causing cellular metabolic regulation. Moreover, a majority of up-regulated genes that are exposed to As(III) are involved in the elimination of ROS (Parvatiyar et al., 2005; Cleiss-Arnold et al., 2010). Bacterial mutants deficient in cellular antioxidants or ROS-scavenging enzymes are often more sensitive to As than wild-type bacteria (Parvatiyar et al., 2005).

In mammals and plants, As(III) exposure additionally produces reactive nitrogen species (RNS), including nitric oxide (NO) and its derivatives (Lynn et al., 1998; Shi et al., 2004; Rao et al., 2011; Leterrier et al., 2012). NO is known to play important roles in living organisms as a defense mechanism and as a messenger that can directly and reversibly activate latent, readily available oxidative stress defense system(s) (Gusarov and Nudler, 2005). However, as the important species of RNS, when NO is overproduced, it will increase nitration, which could deactivate and damage specific nucleic acids, proteins, and lipids, known as nitrosative stress (Leterrier et al., 2012). Nevertheless, little information is available about As induced RNS in bacteria.

Although bacteria have been shown to have special As detoxification pathways, enzymes, and transporters, their metabolic processes are still adversely affected. For example, a microarray analysis shows that approximately 14% of the whole *Herminiimonas asoxydans* genome is significantly affected by 15 min of As(III) exposure (Cleiss-Arnold et al., 2010). Though numerous studies have explored the profiling analysis of the whole organism’s metabolic pathways in response to As stress in plants and animals (Zheng et al., 2005; Yu et al., 2012; Lafuente et al., 2014); the biochemical and molecular mechanisms of As-induced cytotoxicity in bacteria and bacterial responses to that cytotoxicity remain largely unknown. However, understanding the As resistance of bacteria is crucial because of the increasing As contamination in the environment.

LSJC7 is a Gram-negative strain isolated from an antimony tailing, generally contained an As composition of 0.04% (Liu et al., 2010; Su et al., 2012). The genome analysis has revealed that it contains 4,428 protein-coding genes, in which there are two As resistant operons (*arsRDABC* and *arsRBC*; Su et al., 2012). Our previous studies have shown that it has high resistance to many heavy metals and metalloid, especially to As(V) (Chen et al., 2015). Therefore, further investigation is required to understand the toxicity and adaptive responses of LSJC7 to As(V) stress.

Genome-wide transcriptome is an effective method for studying the toxicity and adaptive response pathways by identifying and linking the resulting perturbations (Kaur et al., 2006; Pillai et al., 2014). In this study, we exploited the global transcriptomic profiling to understand whole molecular perturbations and cellular pathways in response to As(V) stress in LSJC7. This enables us to obtain a more detailed picture of the processes affected by As. This will open up for exploration the understanding of the molecular regulating mechanisms of other heavy metal resistant bacteria and to understand the As mediated antibiotic resistance in bacteria.

## Materials and Methods

### Bacterial Strain and Growth Medium

A single clone of Enterobacteriaceae strain LSJC7 was identified with 16S rRNA and grown with LB medium (pH 7.0) overnight at 30°C and 220 rpm (Su et al., 2012), then it was diluted 1:100 with 100 ml of LB medium with/without Na_3_AsO_4_ at 4 mM final concentration in a 250 ml triangular flask as As treatment/control. Meanwhile, a medium control, containing 100 ml of LB medium and Na_3_AsO_4_ but no bacterium, was set up. Each group had three replicates and was incubated in the same conditions overnight as described above. The concentration of LSJC7 was measured using optical density at 600 nm (OD_600_).

### As Speciation Analysis

After LSJC7 was inoculated for 6, 12, and 24 h, 30 ml culture was taken from each triangular flask and centrifuged at 6000 rpm for 5 min. Then 2 ml of liquid supernatant were taken as As speciation samples of medium and bacterial cells were rinsed with deionized water and ice-cold phosphate buffer [1 mM K_2_HPO_4_, 5 mM MES, and 0.5 mM Ca(NO_3_)_2_] for 10 min to remove apoplastic As. Freeze-dried cells and supernatants from medium were kept in the freezer at -80°C until As speciation analysis. Cells were incubated with 10 ml of 1% HNO_3_ overnight, then extraction was performed using 95°C water bath for 60 min with shaking every 10 min (Raber et al., 2012). After extracts cooled to room temperature, aliquots of 1 ml each were filtered through 0.22 μm nylon filter and used directly for HPLC-ICP-MS (7500a; Agilent Technologies) analysis as described previously (Wang et al., 2014). Supernatants from medium were diluted about 2,000-fold with Milli-Q water and filtered through 0.22 μm nylon filter and used directly for HPLC-ICP-MS analysis.

### Total RNA Extraction and RNA-Seq

Both arsenate exposure and control LSJC7 cells were collected at OD_600_ = 0.5 for RNA extraction. Samples (50 ml each) were collected by centrifugation (8,000 × *g*, 10 min). Then the cell pellets were resuspended in 1 ml TRIzol Reagent (Invitrogen, Carlsbad, CA, USA). Two-hundred microliters of chloroform was added to homogenized cells and was mixed well for 30 s and then was incubated at room temperature for 10 min. The aqueous phase was recovered after centrifugation at 12, 000 × *g* for 20 min at 4°C. Twofold of cold ethanol was added to aqueous phase and inverted gently, then incubated at -80°C for 30 min. The precipitated RNA was pelleted by centrifugation for 20 min at 12 000 × *g*. The RNA pellet was washed twice with cold 75% ethanol and then left to air dry. The RNA pellet was resuspended in 30 μl DEPC water. The RNA quantity was assessed using RNA 6000 Nano LabChip Kit in microcapillary electrophoresis (Agilent 2100 Bioanalyzer, Agilent Technologies, Australia). The RNA integrity number (RIN) above 8.0 was used for sequencing. Amount of 3 μg total RNA for each sample was used as input material for the RNA sequencing sample preparations. Sequencing libraries were generated using NEBNext Ultra (NEB, USA). Directional RNA Library Prep Kit for Illumina (NEB) was used following manufacturer’s recommendations and index codes were added to attribute sequences to each sample. Briefly, mRNA was purified from total RNA using poly-T oligo-attached magnetic beads. Fragmentation was carried out using divalent cations under elevated temperature in NEBNext First Strand Synthesis Reaction Buffer (NEB). First strand cDNA was synthesized using random hexamer primer and M-MuLV Reverse Transcriptase (RNaseH-) and second strand cDNA synthesis was subsequently performed using DNA Polymerase I and RNase H. In the reaction buffer, dNTPs with dTTP were replaced by dUTP. Remaining overhangs were converted into blunt ends via exonuclease/polymerase activities. After adenylation of 3’ ends of DNA fragments, NEBNext Adaptors with hairpin loop structure were ligated to prepare for hybridization. In order to select cDNA fragments of preferentially 150∼200 bp in length, the library fragments were purified with AMPure XP system (Beckman Coulter, Beverly, MA, USA). Then, 3 μl USER Enzyme (NEB) was used with size-selected, adaptor-ligated cDNA at 37°C for 15 min followed by 5 min at 95°C before PCR. Then PCR was performed with Phusion High-Fidelity DNA polymerase, Universal PCR primers and Index Primer. At last, products were purified using AMPure XP system and library quality was assessed on the Agilent Bioanalyzer 2100 system. The clustering of the index-coded samples was performed on a cBot Cluster Generation System using TruSeq PE Cluster Kit v3-cBot-HS (Illumina, USA) according to the manufacturer’s instructions. After cluster generation, the library preparations were sequenced on an Illumina Hiseq 2000 platform and 100 bp paired-end reads were generated.

### RNA-Seq Reads Analysis and Differential Expression Analysis

Clean data were obtained using in-house perl scripts by removing reads containing adapter, reads containing ploy-N, and low quality reads from raw data of FASTQ format. RNA-Seq reads (clean data) were aligned to the LSJC7 reference genome^2^. Both building index of reference genome and aligning clean reads to reference genome were used in Bowtie2-2.0.6 (Langmead and Salzberg, 2012). HTSeq v0.5.4p3^3^ was used to count the reads numbers mapped to each gene. And then Reads Per Kilobase of exon model per Million mapped reads (RPKM) of each gene was calculated based on the length of the gene and reads count was mapped to this gene (Mortazavi et al., 2008).

The read counts of each sequenced library were adjusted by *edgeR* program package (Robinson et al., 2010) through one scaling normalized factor. Differential expression analysis of two conditions was performed using the *DEGSeq* R package (1.12.0; Anders and Huber, 2010). The *P*-values were adjusted using the Benjamini and Hochberg (1995) method. Corrected *P*-value of 0.005 and log_2_ (Fold change) of 1 were set as the threshold for significantly differential expression.

Gene Ontology (GO) enrichment analysis of differentially expressed genes was implemented by the *GOseq* R package, in which gene length bias was corrected (Young et al., 2010). GO terms with corrected *P*-value less than 0.05 were considered significantly enriched by differentially expressed genes.

### Real Time Quantitative PCR (RT-qPCR)

Seventeen representative genes were selected and analyzed the expression levels via RT-qPCR to validate the RNA-Seq data. The primers were designed using Primer 3 and listed in Supplementary Table S4. One microgram of total RNA, with contamination DNA removed, was reverse-transcribed using random hexamers and SuperScript III (Invitrogen, USA) to synthesis cDNA according to kit instructions. RT-qPCR was performed with cDNA templates prepared from samples collected in RNA-seq experiment, using CFX96 Real-time PCR System (Bio-Rad, USA) with iQTM SYBR Green Supermix in 96-well plates. The reaction mixture was prepared following kit instructions. All reactions were carried out in three technical replicates. 16S rRNA was used as a reference gene to normalize the results. The relative abundance of gene transcripts among the treatment groups was calculated using the 2^-ΔΔ^*^C^*^t^ method (Livak and Schmittgen, 2001).

### Endogenous ROS Detection

DCFH-DA (Beyotime, China) was diluted in phosphate buffer to make 100 μM working solution. Ten milliliters of LSJC7 culture (OD_600_ = 0.5) was pelleted by centrifugation and washed with phosphate buffer and resuspended in 5 ml of DCFH-DA working solution. Two-hundred microliters of the resultant cell suspension samples were transferred to a 96-well black plate and added 0.5 M As(V) to cell suspension to make final concentration of 0, 4, 16, 32 mM. Fluorescence was measured using a SpectraMax M5 Plate Reader (Molecular Devices, USA) with excitation/emission wavelengths of 488/525 nm.

### Endogenous NO Detection

DAF-FM DA (Beyotime, China) was diluted in phosphate buffer to make 10 μM working solution. Ten milliliters of LSJC7 culture (OD_600_ = 0.5) was pelleted by centrifugation and washed with phosphate buffer and resuspended in 5 ml of DAF-FM DA working solution. Two-hundred microliters of the resultant cell suspension samples was transferred to a 96-well black plate and added 0.5 M As(V) to cell suspension to make final concentration of 0, 4, 16, 32 mM. Fluorescence was measured using a SpectraMax M5 Plate Reader (Molecular Devices, USA) with excitation/emission wavelengths of 494/517 nm.

## Results And Discussion

### Global Changes at Transcriptome Level

**FIGURE 1:**
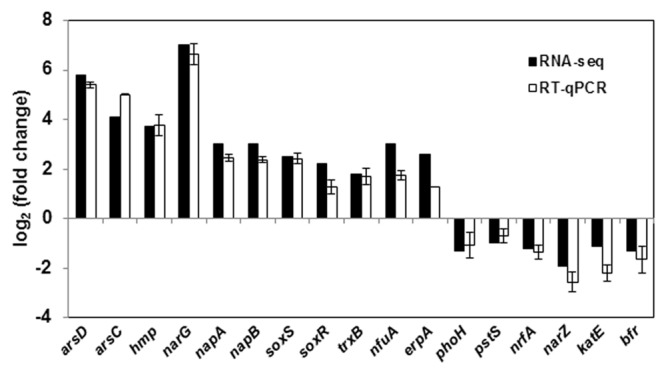
**RT-qPCR validation of 17 genes with significant differential expression in As(V) stress.** RT-qPCR data are mean ± SD from three biological replicates.

**FIGURE 2:**
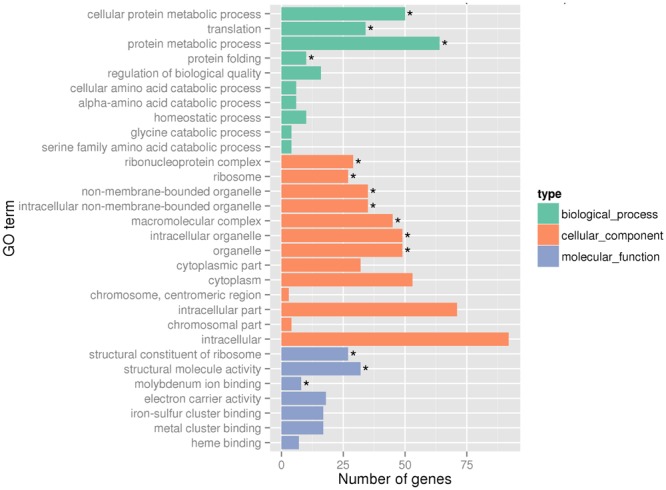
**Gene Ontology (GO) terms enrichment at transcriptome level.** Asterisks represent significantly enriched GO terms (*P* < 0.05).

Global gene expression changes during LSJC7 growth with 4 mM As(V) or without As(V) (control) were analyzed using Illumina RNA deep sequencing (RNA-Seq) technology (van Vliet, 2010) to elucidate molecular mechanisms of As(V) tolerance. To avoid complete suppression of cellular metabolism, 4 mM solution, which was far below the EC50 (33 mM) for LSJC7 exposure to As(V) was chosen as the exposure concentration, and cultures at this concentration showed moderate growth inhibition. Cells in the two treatments were collected at the early exponential phase (OD_600_ was 0.5, **Supplementary Figure S1**). A total of 1.96 and 2.46 G clean bases were obtained from the control and As treatment, respectively. Over 98% of all clean reads aligned to the reference genome (Supplementary Table S2). The RNA-Seq raw reads were deposited to Sequence Read Archive (SRA) database of NCBI with accession numbers SRR3314666 and SRR3314667. Under As(V) exposure, 460 genes showed significant differential expression [log_2_ (Fold Change) > 1 and *q*-value < 0.005 or log_2_ (Fold Change) < -1 and *q*-value < 0.005], in which 285 genes were up-regulated and 175 genes were down-regulated, representing more than 10% of the genome and suggesting a major response of cellular physiology (Supplementary Table S3). Seventeen selected genes, including 11 up-regulated and 6 down-regulated genes on the list of RNA-Seq differentially expressed genes (Supplementary Table S1) were well validated by RT-qPCR (**Figure 1**). GO term enrichment differential expression genes were shown in **Figure 2**. Biological pathways with significant changes were those involved in protein metabolic process, protein folding, translation, amino acid metabolism, homeostatic process, nitrogen metabolism, and carbon metabolism. Molecular functions with significant changes were those involved in structural molecule activity, structural constituent of ribosome, molybdenum ion binding, electron carrier activity, iron–sulfur cluster binding, metal cluster binding, and heme binding.

### Genes Related to As(V) Influx Pumps

Previous report showed that the uptake of arsenate into cells occurs via inorganic phosphate carriers, because As(V) is an analog of inorganic phosphate (Elias et al., 2012). This is consistent with our findings in this study. Our RNA-seq results showed that some genes related to inorganic phosphate transport, including *pstS* and *phoH*, were down-regulated (Supplementary Table S1) under As(V) exposure. The same expression profile was also shown by RT-qPCR (**Figure 1**). The *pstS* gene, encoding a high affinity phosphate binding protein, was down-regulated by 2.1-fold, which might decrease the efficiency of the whole gene cluster *pstSCAB* by limiting the uptake of arsenate (Dyhrman and Haley, 2006). Similarly, *phoH*, encoding protein PhoH which is also named phosphate starvation-inducible protein PsiH, was down-regulated by 2.4-fold. PhoH contains an ATP-binding motif and actually binds to ATP and is bound by PhoB protein, the transcriptional activator of the *pho* regulon, which regulates genes controlling phosphate mobilization, uptake, and metabolism, but its function *in vivo* is not known (Kim et al., 1993). It was suspected that the down-regulation of *phoH* resulted in the decrease of arsenate uptake by the regulation of the *pho* regulon. However, the expression of another phosphate transporter gene *pitA* (low-affinity inorganic phosphate transporter 1) did not change significantly in this study, indicating that PitA was either a constitutive expression or not the main arsenate transporter in LSJC7. These results suggest that reduced expression of influx pumps might be the strategy of LSJC7 to decrease the uptake of As(V).

### As(V) Is Reduced and Extruded as As(III) by LSJC7

**FIGURE 3:**
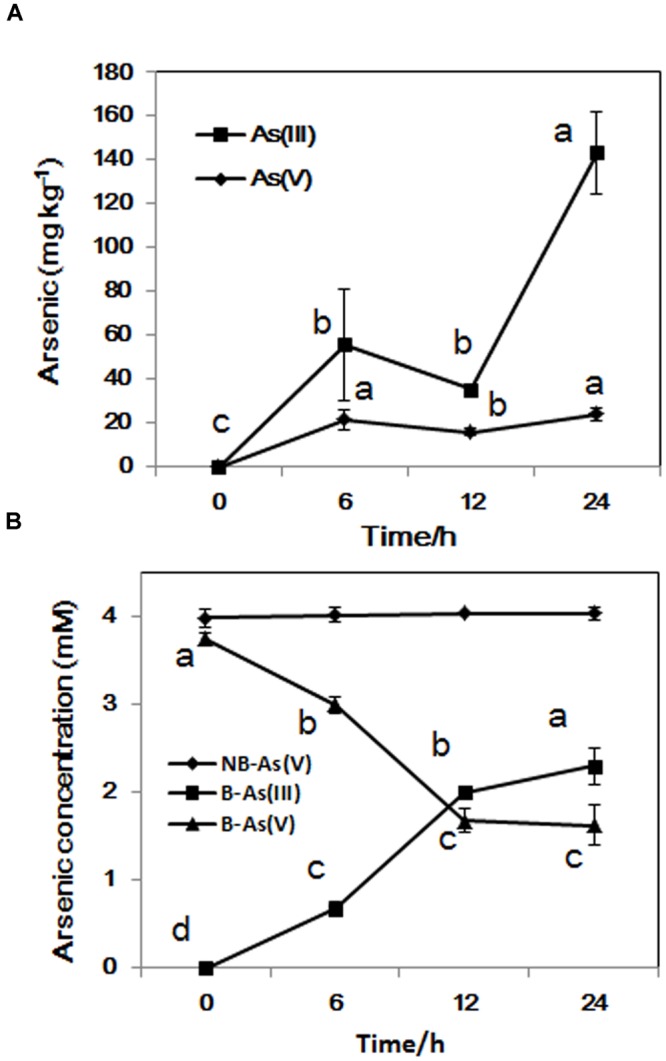
**(A)** As accumulated in LSJC7 cells; **(B)** Fresh LSJC7 cell culture **(B)** and medium control (NB, without LSJC7 cells) were exposed to 4 mM As(V) in LB medium to investigate As(V) reduction. Results are shown as means of three replicates. Different lowercase letters indicate statistically significant difference at *p* < 0.05.

The concentration of intracellular As(III) increased significantly from exponential phase to stationary phase, while the concentration of As(V) stayed at about 20 mg/kg during the whole incubation time (**Figure 3A**). From sampling time point 6 to 24 h, the proportion of As(III) increased from 18 to 58% in the medium, In contrast, the concentration of As(V) in control sample set did not change during the whole process (**Figure 3B**). These results suggest that LSJC7 can reduce As(V) to As(III) quickly and extrude As(III) out of the cell. These reactions should be mediated by *arsRDABC* and *arsRBC* operons in LSJC7. Under As(V) exposure, the transcriptions of genes from the two *ars* operons were up-regulated by 4.6- to 56.4-fold (Supplementary Table S1 and **Figure 1**). The *arsRBC* operon encodes three proteins: ArsR, ArsB, and ArsC. ArsR regulates expression of the chromosomal *ars* operon (Lin et al., 2007). ArsC first converts As(V) to As(III) as an arsenate reductase and ArsB then extrudes As(III) as an As(OH)_3_/H^+^ antiporter, conferring resistance (Lin et al., 2007). ArsC can use reduced thioredoxin (Trx) or glutaredoxin (Grx) to convert As(V) to As(III), namely Trx-linked ArsC or Grx-linked ArsC. In our study, genes encoding the Trx system, including *trxA, trxC, nrdH*, and *trxB* were up-regulated by 2.8- to 21.3-fold in LSJC7 exposed to As(V), suggesting the Trx-linked ArsC in LSJC7. The Trx system, composed of thioredoxin reductase (TrxB encoded by *trxB* in our study), Trx (Thio encoded by *trxA*, Thio2 encoded by *trxC* and NrdH encoded by *nrdH* in our study), and NADPH is known to play critical roles in the redox reaction regulation (Lu et al., 2007), either through redox signaling or more directly as electron donors to the numerous members of the Prx family (Dietz, 2003). The *arsRDABC* operon encodes two additional proteins, ArsA forming a complex with ArsB that catalyzes ATP-driven As(III) eﬄux and ArsD which is a metallochaperone that delivers As(III) to ArsA ATPase. ArsA and ArsD increased As(III) eﬄux and resistance in the environment. The *ars* operon has been found in nearly every sequenced bacterial genome, of which more than 400 bacterial and archaeal *ars* operons containing *arsA* and *arsD* genes have been identified (Ajees et al., 2011). Specially, LSJC7, having two *ars* operons in its genome may remarkably enhance As(V) resistance.

### Genes Related to Nitrate Respiration

Nitrate reductase activity in bacteria could be increased by many environmental stress factors, including hypoxia, NO, and silver nanoparticles, because nitrate acts as the effective alternate terminal electron acceptor after molecular oxygen (Du et al., 2012; Eoh and Rhee, 2013). LSJC7 possesses three nitrate reductases encoded by *narLXKGHJI, narZYWV*, and *napFDAGHBC*. Transcriptome results showed that genes in *narLXKGHJI* and *napFDAGHBC* operon were dramatically up-regulated 24.4 to 138.5-fold and 2.7 to 8.1-fold, respectively, *narZ* and *narY* were down-regulated about fourfold (Supplementary Table S1 and **Figure 1**). Meanwhile, many of the genes implicated in aerobic respiration (TCA cycle), such as *sdhB* and *frdA*, were down-regulated around twofold under As stress (Supplementary Table S1). From these results, we suspected that LSJC7 might prefer to use anaerobic nitrate respiration for energy production under As(V) stress.

On the other hand, nitrate respiration also could accumulate nitrite *in vivo*. In *mycobacteria*, export of the nitrite, a toxic by-product of nitrate respiration, by NarK3 and NarU is the main protection strategy (Tan et al., 2010). Under As stress, 138.5-fold up-regulated *narK* gene might indicate the export of nitrite by NarK was important part of the protection strategy. Also consistent with previous report that NirBD is required to reduce nitrite to ammonia in *Enterobacteria* (Cole, 1996), *nirB* was also up-regulated by 2.3-fold under As(V) stress in our study. It has been also demonstrated that low concentrations of NO are generated as a by-product of this metabolism (Corker and Poole, 2003; Spiro, 2007).

### Endogenous NO Up-regulated *nsrR* and *hmp*

**FIGURE 4:**
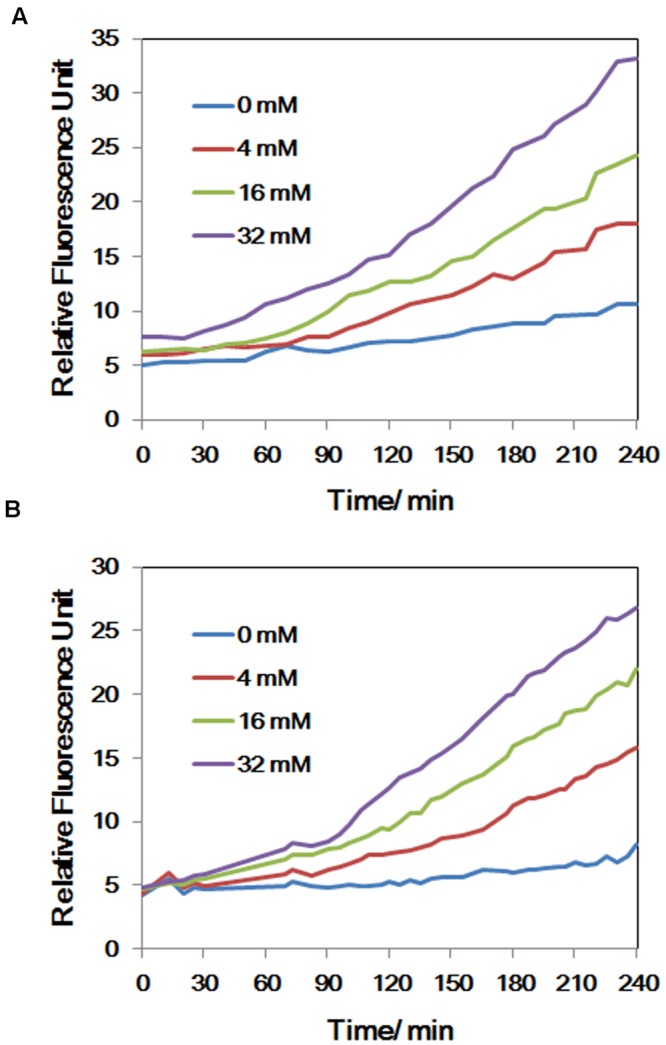
**(A)** The concentrations of NO of LSJC7 exposure to 0, 4, 16, and 32 mM As; **(B)** The concentrations of ROS of LSJC7 exposure to 0, 4, 16, and 32 mM As. Results are shown as means of three replicates.

Intracellular NO production was detected in As(V) stressed LSJC7 cells using a DAF-FM DA probe. The concentration of NO increased with the As(V) concentration in the medium when LSJC7 was exposed to 0, 4, 16, and 32 mM As (**Figure 4A**). Consistently, genes encoding proteins of NO regulon (NsrR) and detoxification (Hmp and NrfA) were also regulated when LSJC7 was exposed to 4 mM As (Supplementary Table S1). NO can be generated via nitrite reduction by enzymatic or non-enzymatic mechanisms in Gram-negative bacteria (Puyaubert and Baudouin, 2014). NsrR is a global transcription repressor whose activity is responding to NO. Targets for NsrR regulation are at least 30 genes, including *hmp, nrfA, ytfE, hcp-hcr, napF, napD, napH*, and *napB* (Filenko et al., 2007). Flavohemoprotein (also named Flavohemoglobin, Hmp) is involved in conversion of NO to nitrate with substantial O_2_ consumption (Stevanin et al., 2000). The *hmp* genes were dramatically up-regulated by 13.3-fold, which suggested high concentration of NO was harmful to cells and strongly needed to be detoxified. Cytochrome c552 (NrfA), encoded by the *nrfA* gene, acts as a nitrite reductase and is also responsible for NO detoxification (Pittman et al., 2007). The *nrfA* gene is negatively regulated by NsrR and down-regulated by 2.1-fold (Supplementary Table S1 and **Figure 1**), suggesting NO detoxification regulated by *hmp* to generate nitrate may provide more benefits to LSJC7. Our data suggested that NO was generated under As(V) stress, while regulation system of NO was also activated to control its homeostasis and against nitrosative stress.

### ROS Generation under As(V) Stress

To demonstrate that ROS were produced in LSJC7 under As(V) stress, we examined the intracellular concentration of ROS of LSJC7 exposed to 0, 4, 16, and 32 mM As(V). As expected, the concentration of ROS increased with increasing As(V) concentrations in the medium and the treatment time (**Figure 4B**). It has been reported that ROS played critical roles in bacterial cell death from antibiotics, hydroxyurea, and silver stress (Davies et al., 2009; Morones-Ramirez et al., 2013; Dwyer et al., 2014). ROS mainly interacts with thiols, metal centers, nucleotide bases, protein tyrosines, and lipids (Fang, 2004) and these interactions result in damages to nucleic acids, proteins, and lipids, thus affecting normal cellular functions (Yang et al., 2007; Ray et al., 2012). In our transcriptome results, many repair genes were up-regulated robustly (Supplementary Table S1), which might indicate that ROS induced by As(V) would be an important reason for cellular damage. However, gene encoding superoxide dismutase (SOD), which directly nullifies ROS, was not found up-regulated in As(V) stressed cells. Moreover, genes encoding Prxs, including *osmC, katG, katE*, and *cpo*, were down-regulated by 2.1 to 3.1-fold in LSJC7 exposed to As(V) (Supplementary Table S1 and **Figure 1**). These results suggested that alternative antioxidant systems might be used against ROS under As(V) stress.

### Genes Related to Fe–S Clusters Biogenesis and Iron Recruiting

Reactive oxygen species can directly affect IscR, which modulates a set of genes involved in iron–sulfur prosthetic groups (Fe–S clusters) biogenesis (namely, the Isc and Suf systems as well as ErpA and NfuA; Blanchard et al., 2007; Py and Barras, 2010). RNA-seq results showed that genes *iscR, erpA*, and *nfuA* were up-regulated by 3.6 to 8.2-fold in As(V) stressed cells (Supplementary Table S1). Meanwhile, the expression of *erpA* and *nfuA* was also validated by RT-qPCR (**Figure 1**). These results indicate increased Fe–S cluster biogenesis. Many genes encoding proteins containing Fe–S clusters (Fe–S proteins) were regulated in As(V) stressed cells; most of them were up-regulated. These proteins have prominent roles in multiple important cellular processes, including respiration (NapF, NapA, NapG, NapH, NarG, NarH encoded by *napF, napA, napG, napH, narG, narH*, and NarY, NarZ encoded by *narY, narZ*), central metabolism (FhuF, BfD, Hcp, Hcr encoded by *fhuF, bfd, hcp, hcr*), biosynthetic pathways (NfuA, ErpA encoded by *nfuA, erpA*), and gene regulation (NsrR, IscR, SoxR encoded by *nsrR iscR, soxR*). The robust regulation of these Fe–S proteins could be the reason of increased Fe–S clusters biogenesis. In As(V) stressed cells more iron should be recruited to alleviate the stress. Consistent with this, genes encoding proteins related to iron uptake were regulated under As(V) exposure (Supplementary Table S1); *feoA* encoding FeoA for ferrous iron uptake (Lau et al., 2013; Weaver et al., 2013), *fhuA, fepD*, and *fhuF* encoding FhuA, FepD, and FhuF for ferric iron (siderophore) uptake (Chenault and Earhart, 1992; Cleiss-Arnold et al., 2010; Miethke, 2013), were up-regulated by 3.0 to 7.7-fold; *exbB* encoding ExbB for the energy-transducing TonB–ExbB–ExbD complex of ferric iron transport (Andrews et al., 2003) was up-regulated 5.3-fold. Genes *bfd* and *bfr*, encoding iron storage protein bacterioferritin-associated ferredoxin (Bfd) and bacterioferritin (Bfr), are reciprocally regulated by iron availability via ferric uptake regulator (Quail et al., 1996). Bfd and Bfr can store iron as ferric iron to protect cells against ROS resulting from ferrous iron overload (Andrews et al., 2003). In RNA-seq results, *bfd* was up-regulated by 22.8-fold, while *bfr* was down-regulated by 2.4-fold (Supplementary Table S1). The regulations of these iron related genes indicated As(V) stressed cells robustly recruited iron to cells.

### Genes Related to Iron–Sulfur Cluster Repair and Suppressed Fenton Reaction

The gene *ytfE*, known as one of NsrR’s targets (Filenko et al., 2007; Spiro, 2007), encoding Fe–S cluster repair protein YtfE, was significantly up-regulated, by 72.2-fold (Supplementary Table S1), suggesting that robust repair occurred in damaged Fe–S clusters in As(V) stressed cells. Fe–S clusters in dehydratases can be damaged by univalent oxidants, converting the clusters to inactive form and causing concomitant loss of Fe^2+^ which fuels Fenton reaction to produce the highly reactive HO^∙^ (Djaman et al., 2004; Jang and Imlay, 2007; Py and Barras, 2010). Fe^2+^ is recruited and integrated to Fe–S clusters by YtfE, which could decrease the cascade of the Fenton reaction (Py and Barras, 2010). The H_2_O_2_ scavenging system was another critical component to decrease Fenton reaction. In our study, the *hcp–hcr* genes, also modulated by NsrR, which encodes a hybrid cluster protein Hcp and its redox partner Hcr, were up-regulated under As(V) stress. Hcp–Hcr complex has been suggested to be a hydroxylamine reductase or peroxidase (Wolfe et al., 2002; Almeida et al., 2006). This result suggested that Hcp–Hcr might play an important role in scavenging H_2_O_2_ in As(V) stressed cells. Taken together, NO could induce Fe–S cluster repair and suppress Fenton reaction by modulating the expression of *nsrR*.

### Genes Related to DNA and Protein Repair

In our study, the transcriptions of *soxR* and *soxS* were highly up-regulated under As(V) exposure (Supplementary Table S1 and **Figure 1**). These two genes encode two separate transcription activators SoxR and SoxS that participate in a two-step activation (Pomposiello and Demple, 2001). SoxR is a transcription factor containing [2Fe–2S] clusters, which coordinate the response to oxidative stress (Lushchak, 2011). SoxR can be distinctly activated by superoxide stress and thereby induce SOD in aerobic stress (Pomposiello and Demple, 2001; Gu and Imlay, 2011). However, in anaerobic cells, SoxR is able to be activated by redox drugs when alternative respiratory acceptors were provided (Gu and Imlay, 2011). SoxR also exhibits a mechanism of activation by NO, which is independent of the presence of oxygen (Pomposiello and Demple, 2001). In *E. coli*, SoxRS modulates more than 100 genes, encoding various proteins (Blanchard et al., 2007), which have diverse biological functions; including NADPH regeneration, superoxide scavenging, DNA repair, protein repair, recycling of damaged macromolecules, xenobiotic eﬄux, and carbon metabolism (Pomposiello and Demple, 2001; Blanchard et al., 2007). We suggest that the LSJC7 SoxRS gene, at least, could modulate DNA and protein repair in As(V) stressed cells, though more studies should be done to illuminate its targets. Because As(V) stressed cells were undergoing a hypoxia circumstance and the gene encoding SOD was not found significantly modulated, we propose that NO was the reason to activate SoxRS in LSJC7.

Transcriptome data showed that genes involved in both DNA and protein repair were modulated, indicating As(V) induced oxidative stress occurring. Nucleic acids could be damaged by Fenton chemistry, via the formation of either iron-oxo intermediates or HO^∙^ (Dwyer et al., 2014). The Gene *smrA*, encoding Smr protein (MutS2) which is a DNA repair enzyme (Fukui and Kuramitsu, 2011), was up-regulated by 2.1-fold under As stress (Supplementary Table S1). MutS2 has an endonuclease domain located in the C-terminal region also called the small MutS-related (Smr) domain (Moreira and Philippe, 1999). MutS is also found overexpressed in antibiotic-induced redox stress, which limits the antibiotic lethality (Dwyer et al., 2014). This result indicated that As(V) stress might induce DNA damage, however, cells could produce special proteins to resist or repair that damage.

Genes involved in protein turnover were also modulated in As(V) stress. Three out of the six genes encoding proteases were up-regulated, the other three were down-regulated, and 53 genes encoding proteins for protein synthesis and folding were particularly up-regulated (Supplementary Table S1). In our present study, genes of more than 10% of the genome showed significant differential expression under As stress, which must induce significant protein turnover. These results suggested vast scale protein synthesis and degradation processes under As(V) stress, which could repair the damaged proteins and produce new proteins for handling As(V) stress. Similar observations are also found with transition metals, including Mn(II), Fe(II), Co(II), Ni(II), Cu(II), and Zn(II) (Kaur et al., 2006), suggesting that protein turnover might be a general strategy to withstand metal(loid) stress.

**FIGURE 5:**
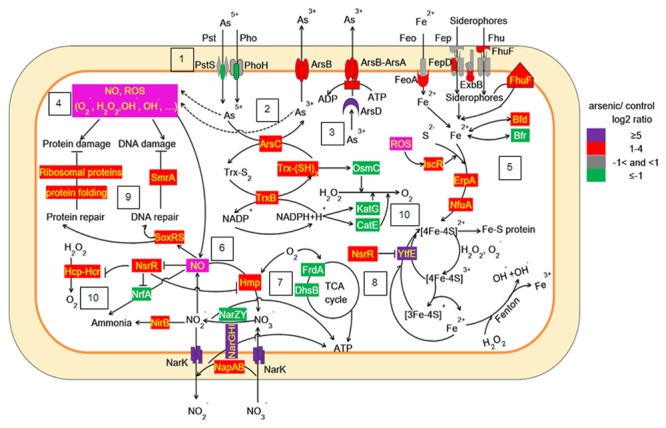
**Proposed scheme illustrating the toxicity and adaptive-response pathways of LSJC7 to As(V) stress.** (1) As(V) influx pumps was down-regulation; (2) ArsC converted As(V) to As(III); (3) ArsB extruded As(III); (4) Arsenic induced ROS and NO generation; (5) ROS induced Fe–S clusters biogenesis and iron recruiting; (6) Endogenous NO up-regulated *nsrR* and *hmp*; (7) Genes related to nitrate respiration and TCA cycle; (8) NO induced Iron–sulfur cluster repair and suppressed Fenton reaction; (9) SoxRS activated by NO might modulate DNA and protein repair; (10) Peroxiredoxins were modulated.

**FIGURE 6:**
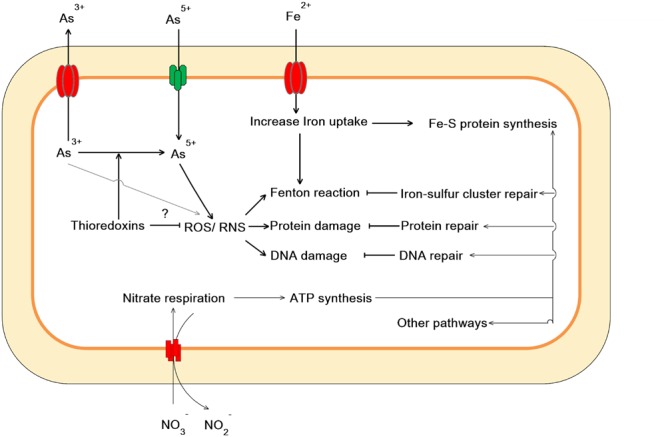
**Proposed scheme illustrating cellular events related to As(V) stress in LSJC7**.

Taken together, our work showed that As(V) disturbed multiple cellular networks and processes of LSJC7 (**Figures 5** and **6**). To decrease the intracellular As accumulation, As(V) was reduced to As(III) and As(III) was expelled into medium. However, the transformation between As(V) and As(III) induced cascades of redox reactions in the cell and changed the cellular redox state and As(III) can strongly interact with vicinal thiols such as cysteine pairs in proteins (Hughes, 2002). Moreover, various types of reactive species, such as ROS and NO, could be involved in the damage of proteins and DNA. However, the expression of specific genes encoding transcriptional regulators, such as *nsrR* and *soxRS*, was increased under As stress. NsrR modulated the expression of several genes including *hmp, nrfA, hcp–hcr*, and *ytfE*, which were responsible for NO detoxification, H_2_O_2_ scavenging, and Fe–S clusters repair. Moreover, SoxRS in LSJC7 could modulate DNA and protein repair in As(V) stressed cells.

## Conclusion

Enterobacteriaceae strain LSJC7 has evolved multiple strategies to survive As stress. It not only actively decreases cellular As concentration, but also launches an oxidative stress defense system to prevent cells from damages induced by As and reactive species, and repair some unavoidable cell damages. The same strategies could be employed by most Gram-negative bacteria.

## Author Contributions

JY, Y-GZ, and J-QS conceived and designed the project. YZ, SC, and XH did the transcriptomic experiments. XX and YY performed the arsenic speciation analysis. YZ and XH analyzed the data. YZ, SC, Y-GZ and JY wrote the manuscript. All authors read and approved the final manuscript.

## Conflict of Interest Statement

The authors declare that the research was conducted in the absence of any commercial or financial relationships that could be construed as a potential conflict of interest.

## Footnotes

1 http://www.atsdr.cdc.gov/SPL/index.html

2 http://www.ncbi.nlm.nih.gov/nuccore/AMFN00000000

3 https://pypi.python.org/pypi/HTSeq

## Supplementary Material

FIGURE S1 **Growth rates of LSJC7 under 4 mM As(V) stress.** The arrows represent the sampling time for transcriptome, and the boxes represent the sampling time for As species analysis.

## References

[B1] Ajees A. A. Yang J. Rosen B. P. (2011). The ArsD As(III) metallochaperone. *Biometals* 24 391–399. 10.1007/s10534-010-9398-x 21188475 PMC3773603

[B2] Almeida C. C. Romao C. V. Lindley P. F. Teixeira M. Saraiva L. M. (2006). The role of the hybrid cluster protein in oxidative stress defense. *J. Biol. Chem.* 281 32445–32450. 10.1074/jbc.M605888200 16928682

[B3] Anders S. Huber W. (2010). Differential expression analysis for sequence count data. *Genome Biol.* 11:R106 10.1186/gb-2010-11-10-r106 PMC3218662 20979621

[B4] Andrews S. C. Robinson A. K. Rodriguez-Quinones F. (2003). Bacterial iron homeostasis. *FEMS Microbiol. Rev.* 27 215–237. 10.1016/S0168-6445(03)00055-X 12829269

[B5] Benjamini Y. Hochberg Y. (1995). Controlling the false discovery rate-a practical and powerful approach to multiple testing. *J. R. Stat. Soc. Ser. B Methodol.* 57 289–300.

[B6] Blanchard J. L. Wholey W. Y. Conlon E. M. Pomposiello P. J. (2007). Rapid changes in gene expression dynamics in response to superoxide reveal SoxRS-dependent and independent transcriptional networks. *PLoS ONE* 2:e1186 10.1371/Journal.Pone.0001186 PMC2064960 18000553

[B7] Chen S. Li X. Sun G. Zhang Y. Su J. Ye J. (2015). Heavy metal induced antibiotic resistance in bacterium LSJC7. *Int. J. Mol. Sci.* 16 23390–23404. 10.3390/ijms161023390 26426011 PMC4632705

[B8] Chenault S. S. Earhart C. F. (1992). Identification of hydrophobic proteins FepD and FepG of the *Escherichia coli* ferrienterobactin permease. *J. Gen. Microbiol.* 138 2167–2171. 10.1099/00221287-138-10-2167 1479347

[B9] Cleiss-Arnold J. Koechler S. Proux C. Fardeau M. L. Dillies M. A. Coppee J. Y. (2010). Temporal transcriptomic response during arsenic stress in *Herminiimonas arsenicoxydans*. *BMC Genomics* 11:709 10.1186/1471-2164-11-709 PMC3022917 21167028

[B10] Cole J. (1996). Nitrate reduction to ammonia by enteric bacteria: redundancy, or a strategy for survival during oxygen starvation? *FEMS Microbiol. Lett.* 136 1–11. 10.1111/j.1574-6968.1996.tb08017.x 8919448

[B11] Corker H. Poole R. K. (2003). Nitric oxide formation by *Escherichia coli*. Dependence on nitrite reductase, the NO-sensing regulator Fnr, and flavohemoglobin Hmp. *J. Biol. Chem.* 278 31584–31592. 10.1074/jbc.M303282200 12783887

[B12] Cullen W. R. Reimer K. J. (1989). Arsenic speciation in the environment. *Chem. Rev.* 89 713–764. 10.1021/cr00094a002

[B13] Davies B. W. Kohanski M. A. Simmons L. A. Winkler J. A. Collins J. J. Walker G. C. (2009). Hydroxyurea induces hydroxyl radical-mediated cell death in *Escherichia coli*. *Mol. Cell* 36 845–860. 10.1016/j.molcel.2009.11.024 20005847 PMC2821657

[B14] Dietz K. J. (2003). Plant peroxiredoxins. *Annu. Rev. Plant Biol.* 54 93–107. 10.1146/annurev.arplant.54.031902.134934 14502986

[B15] Djaman O. Outten F. W. Imlay J. A. (2004). Repair of oxidized iron-sulfur clusters in *Escherichia coli*. *J. Biol. Chem.* 279 44590–44599. 10.1074/jbc.M406487200 15308657

[B16] Du H. Lo T. M. Sitompul J. Chang M. W. (2012). Systems-level analysis of *Escherichia coli* response to silver nanoparticles: the roles of anaerobic respiration in microbial resistance. *Biochem. Biophys. Res. Commun.* 424 657–662. 10.1016/j.bbrc.2012.06.134 22771582

[B17] Dwyer D. J. Belenky P. A. Yang J. H. MacDonald I. C. Martell J. D. Takahashi N. (2014). Antibiotics induce redox-related physiological alterations as part of their lethality. *Proc. Natl. Acad. Sci. U.S.A.* 111 E2100–E2109. 10.1073/pnas.1401876111 24803433 PMC4034191

[B18] Dyhrman S. T. Haley S. T. (2006). Phosphorus scavenging in the unicellular marine diazotroph *Crocosphaera watsonii*. *Appl. Environ. Microbiol.* 72 1452–1458. 10.1128/AEM.72.2.1452-1458.2006 16461699 PMC1392970

[B19] Elias M. Wellner A. Goldin-Azulay K. Chabriere E. Vorholt J. A. Erb T. J. (2012). The molecular basis of phosphate discrimination in arsenate-rich environments. *Nature* 491 134–137. 10.1038/nature11517 23034649

[B20] Eoh H. Rhee K. Y. (2013). Multifunctional essentiality of succinate metabolism in adaptation to hypoxia in *Mycobacterium tuberculosis*. *Proc. Natl. Acad. Sci. U.S.A.* 110 6554–6559. 10.1073/pnas.1219375110 23576728 PMC3631649

[B21] Fang F. C. (2004). Antimicrobial reactive oxygen and nitrogen species: concepts and controversies. *Nat. Rev. Microbiol.* 2 820–832. 10.1038/nrmicro1004 15378046

[B22] Filenko N. Spiro S. Browning D. F. Squire D. Overton T. W. Cole J. (2007). The NsrR regulon of *Escherichia coli* K-12 includes genes encoding the hybrid cluster protein and the periplasmic, respiratory nitrite reductase. *J. Bacteriol.* 189 4410–4417. 10.1128/JB.00080-07 17449618 PMC1913375

[B23] Fukui K. Kuramitsu S. (2011). Structure and function of the small MutS-related domain. *Mol. Biol. Int.* 2011 691735 10.4061/2011/691735 PMC3200294 22091410

[B24] Gu M. Imlay J. A. (2011). The SoxRS response of *Escherichia coli* is directly activated by redox-cycling drugs rather than by superoxide. *Mol. Microbiol.* 79 1136–1150. 10.1111/j.1365-2958.2010.07520.x 21226770 PMC3071027

[B25] Gusarov I. Nudler E. (2005). NO-mediated cytoprotection: instant adaptation to oxidative stress in bacteria. *Proc. Natl. Acad. Sci. U.S.A.* 102 13855–13860. 10.1073/pnas.0504307102 16172391 PMC1236549

[B26] Harrison J. J. Tremaroli V. Stan M. A. Chan C. S. Vacchi-Suzzi C. Heyne B. J. (2009). Chromosomal antioxidant genes have metal ion-specific roles as determinants of bacterial metal tolerance. *Environ. Microbiol.* 11 2491–2509. 10.1111/j.1462-2920.2009.01973.x 19555372

[B27] Hughes M. F. (2002). Arsenic toxicity and potential mechanisms of action. *Toxicol. Lett.* 133 1–16. 10.1016/S0378-4274(02)00084-X 12076506

[B28] Jang S. Imlay J. A. (2007). Micromolar intracellular hydrogen peroxide disrupts metabolism by damaging iron-sulfur enzymes. *J. Biol. Chem.* 282 929–937. 10.1074/jbc.M607646200 17102132 PMC5136138

[B29] Kaur A. Pan M. Meislin M. Facciotti M. T. El-Gewely R. Baliga N. S. (2006). A systems view of haloarchaeal strategies to withstand stress from transition metals. *Genome Res.* 16 841–854. 10.1101/gr.5189606 16751342 PMC1484451

[B30] Kim S. K. Makino K. Amemura M. Shinagawa H. Nakata A. (1993). Molecular analysis of the phoH gene, belonging to the phosphate regulon in *Escherichia coli*. *J. Bacteriol.* 175 1316–1324. 8444794 10.1128/jb.175.5.1316-1324.1993 PMC193217

[B31] Lafuente A. Perez-Palacios P. Doukkali B. Molina-Sanchez M. D. Jimenez-Zurdo J. I. Caviedes M. A. (2014). Unraveling the effect of arsenic on the model Medicago-Ensifer interaction: a transcriptomic meta-analysis. *New Phytol.* 205 255–272. 10.1111/nph.13009 25252248

[B32] Langmead B. Salzberg S. L. (2012). Fast gapped-read alignment with Bowtie 2. *Nat. Methods* 9 357–359. 10.1038/nmeth.1923 22388286 PMC3322381

[B33] Lau C. K. Ishida H. Liu Z. Vogel H. J. (2013). Solution structure of *Escherichia coli* FeoA and its potential role in bacterial ferrous iron transport. *J. Bacteriol.* 195 46–55. 10.1128/JB.01121-12 23104801 PMC3536175

[B34] Lemire J. A. Harrison J. J. Turner R. J. (2013). Antimicrobial activity of metals: mechanisms, molecular targets and applications. *Nat. Rev. Microbiol.* 11 371–384. 10.1038/nrmicro3028 23669886

[B35] Leterrier M. Airaki M. Palma J. M. Chaki M. Barroso J. B. Corpas F. J. (2012). Arsenic triggers the nitric oxide (NO) and S-nitrosoglutathione (GSNO) metabolism in *Arabidopsis*. *Environ. Pollut.* 166 136–143. 10.1016/j.envpol.2012.03.012 22504427

[B36] Lin Y. F. Walmsley A. R. Rosen B. P. (2006). An arsenic metallochaperone for an arsenic detoxification pump. *Proc. Natl. Acad. Sci. U.S.A.* 103 15617–15622. 10.1073/pnas.0603974103 17030823 PMC1622871

[B37] Lin Y. F. Yang J. B. Rosen B. P. (2007). ArsD: an As(III) metallochaperone for the ArsAB As(III)-translocating ATPase. *J. Bioenerg. Biomembr.* 39 453–458. 10.1007/s10863-007-9113-y 17955352

[B38] Liu F. Le X. C. McKnight-Whitford A. Xia Y. Wu F. Elswick E. (2010). Antimony speciation and contamination of waters in the Xikuangshan antimony mining and smelting area, China. *Environ. Geochem. Health* 32 401–413. 10.1007/s10653-010-9284-z 20101438

[B39] Livak K. J. Schmittgen T. D. (2001). Analysis of relative gene expression data using real-time quantitative PCR and the 2(T)(-Delta Delta C) method. *Methods* 25 402–408. 10.1006/meth.2001.1262 11846609

[B40] Lu J. Chew E. H. Holmgren A. (2007). Targeting thioredoxin reductase is a basis for cancer therapy by arsenic trioxide. *Proc. Natl. Acad. Sci. U.S.A.* 104 12288–12293. 10.1073/pnas.0701549104 17640917 PMC1940330

[B41] Lushchak V. I. (2011). Adaptive response to oxidative stress: bacteria, fungi, plants and animals. *Comp. Biochem. Physiol. C Toxicol. Pharmacol.* 153 175–190. 10.1016/j.cbpc.2010.10.004 20959147

[B42] Lynn S. Shiung J. N. Gurr J. R. Jan K. Y. (1998). Arsenite stimulates poly(ADP-ribosylation) by generation of nitric oxide. *Free Radic. Biol. Med.* 24 442–449. 10.1016/S0891-5849(97)00279-7 9438557

[B43] Miethke M. (2013). Molecular strategies of microbial iron assimilation: from high-affinity complexes to cofactor assembly systems. *Metallomics* 5 15–28. 10.1039/c2mt20193c 23192658

[B44] Moreira D. Philippe H. (1999). Smr: a bacterial and eukaryotic homologue of the C-terminal region of the MutS2 family. *Trends Biochem. Sci.* 24 298–300. 10.1016/S0968-0004(99)01419-X 10431172

[B45] Morones-Ramirez J. R. Winkler J. A. Spina C. S. Collins J. J. (2013). Silver enhances antibiotic activity against gram-negative bacteria. *Sci. Transl. Med.* 5:190ra181 10.1126/scitranslmed.3006276 PMC3771099 23785037

[B46] Mortazavi A. Williams B. A. McCue K. Schaeffer L. Wold B. (2008). Mapping and quantifying mammalian transcriptomes by RNA-Seq. *Nat. Methods* 5 621–628. 10.1038/nmeth.1226 18516045 PMC13303166

[B47] Parvatiyar K. Alsabbagh E. M. Ochsner U. A. Stegemeyer M. A. Smulian A. G. Hwang S. H. (2005). Global analysis of cellular factors and responses involved in *Pseudomonas aeruginosa* resistance to arsenite. *J. Bacteriol.* 187 4853–4864. 10.1128/JB.187.14.4853-4864.2005 15995200 PMC1169530

[B48] Pillai S. Behra R. Nestler H. Suter M. J. Sigg L. Schirmer K. (2014). Linking toxicity and adaptive responses across the transcriptome, proteome, and phenotype of *Chlamydomonas reinhardtii* exposed to silver. *Proc. Natl. Acad. Sci. U.S.A.* 111 3490–3495. 10.1073/pnas.1319388111 24550482 PMC3948315

[B49] Pittman M. S. Elvers K. T. Lee L. Jones M. A. Poole R. K. Park S. F. (2007). Growth of *Campylobacter jejuni* on nitrate and nitrite: electron transport to NapA and NrfA via NrfH and distinct roles for NrfA and the globin Cgb in protection against nitrosative stress. *Mol. Microbiol.* 63 575–590. 10.1111/j.1365-2958.2006.05532.x 17241202

[B50] Pomposiello P. J. Demple B. (2001). Redox-operated genetic switches: the SoxR and OxyR transcription factors. *Trends Biotechnol.* 19 109–114. 10.1016/S0167-7799(00)01542-0 11179804

[B51] Puyaubert J. Baudouin E. (2014). New clues for a cold case: nitric oxide response to low temperature. *Plant Cell Environ.* 37 2623–2630. 10.1111/pce.12329 24720833

[B52] Py B. Barras F. (2010). Building Fe-S proteins: bacterial strategies. *Nat. Rev. Microbiol.* 8 436–446. 10.1038/nrmicro2356 20467446

[B53] Quail M. A. Jordan P. Grogan J. M. Butt J. N. Lutz M. Thomson A. J. (1996). Spectroscopic and voltammetric characterisation of the bacterioferritin-associated ferredoxin of *Escherichia coli*. *Biochem. Biophys. Res. Commun.* 229 635–642. 10.1006/bbrc.1996.1856 8954950

[B54] Raber G. Stock N. Hanel P. Murko M. Navratilova J. Francesconi K. A. (2012). An improved HPLC-ICPMS method for determining inorganic arsenic in food: application to rice, wheat and tuna fish. *Food Chem.* 134 524–532. 10.1016/j.foodchem.2012.02.113

[B55] Rao K. P. Vani G. Kumar K. Wankhede D. P. Misra M. Gupta M. (2011). Arsenic stress activates MAP kinase in rice roots and leaves. *Arch. Biochem. Biophys.* 506 73–82. 10.1016/j.abb.2010.11.006 21081102

[B56] Ray P. D. Huang B. W. Tsuji Y. (2012). Reactive oxygen species (ROS) homeostasis and redox regulation in cellular signaling. *Cell. Signal.* 24 981–990. 10.1016/j.cellsig.2012.01.008 22286106 PMC3454471

[B57] Robinson M. D. McCarthy D. J. Smyth G. K. (2010). edgeR: a Bioconductor package for differential expression analysis of digital gene expression data. *Bioinformatics* 26 139–140. 10.1093/bioinformatics/btp616 19910308 PMC2796818

[B58] Shi H. Shi X. Liu K. J. (2004). Oxidative mechanism of arsenic toxicity and carcinogenesis. *Mol. Cell. Biochem.* 255 67–78. 10.1023/B:MCBI.0000007262.26044.e8 14971647

[B59] Spiro S. (2007). Regulators of bacterial responses to nitric oxide. *FEMS Microbiol. Rev.* 31 193–211. 10.1111/j.1574-6976.2006.00061.x 17313521

[B60] Stevanin T. M. Ioannidis N. Mills C. E. Kim S. O. Hughes M. N. Poole R. K. (2000). Flavohemoglobin Hmp affords inducible protection for *Escherichia coli* respiration, catalyzed by cytochromes bo’ or bd, from nitric oxide. *J. Biol. Chem.* 275 35868–35875. 10.1074/jbc.M002471200 10915782

[B61] Su J. Ye J. Zhu Y. (2012). Draft genome sequence of a novel bacterial strain, LSJC7, belonging to the family *Enterobacteriaceae* with dual resistance to arsenic and tetracycline. *J. Bacteriol.* 194 7005–7006. 10.1128/JB.01652-12 23209248 PMC3510629

[B62] Tan M. P. Sequeira P. Lin W. W. Phong W. Y. Cliff P. Ng S. H. (2010). Nitrate respiration protects hypoxic *Mycobacterium tuberculosis* against acid- and reactive nitrogen species stresses. *PLoS ONE* 5:e13356 10.1371/journal.pone.0013356 PMC2965054 21048946

[B63] van Vliet A. H. (2010). Next generation sequencing of microbial transcriptomes: challenges and opportunities. *FEMS Microbiol. Lett.* 302 1–7. 10.1111/j.1574-6968.2009.01767.x 19735299

[B64] Wang F. H. Qiao M. Lv Z. E. Guo G. X. Jia Y. Su Y. H. (2014). Impact of reclaimed water irrigation on antibiotic resistance in public parks, Beijing, China. *Environ. Pollut.* 184 247–253. 10.1016/j.envpol.2013.08.038 24071635

[B65] Weaver E. A. Wyckoff E. E. Mey A. R. Morrison R. Payne S. M. (2013). FeoA and FeoC are essential components of the *Vibrio cholerae* ferrous iron uptake system, and FeoC interacts with FeoB. *J. Bacteriol.* 195 4826–4835. 10.1128/JB.00738-13 23955009 PMC3807486

[B66] Wolfe M. T. Heo J. Garavelli J. S. Ludden P. W. (2002). Hydroxylamine reductase activity of the hybrid cluster protein from *Escherichia coli*. *J. Bacteriol.* 184 5898–5902. 10.1128/JB.184.21.5898-5902.2002 12374823 PMC135376

[B67] Yang P. He X. Q. Peng L. Li A. P. Wang X. R. Zhou J. W. (2007). The role of oxidative stress in hormesis induced by sodium arsenite in human embryo lung fibroblast (HELF) cellular proliferation model. *J. Toxicol. Environ. Health A* 70 976–983. 10.1080/15287390701290832 17479414

[B68] Young M. D. Wakefield M. J. Smyth G. K. Oshlack A. (2010). Gene ontology analysis for RNA-seq: accounting for selection bias. *Genome Biol.* 11:R14 10.1186/gb-2010-11-2-r14 PMC2872874 20132535

[B69] Yu L. J. Luo Y. F. Liao B. Xie L. J. Chen L. Xiao S. (2012). Comparative transcriptome analysis of transporters, phytohormone and lipid metabolism pathways in response to arsenic stress in rice (*Oryza sativa*). *New Phytol.* 195 97–112. 10.1111/j.1469-8137.2012.04154.x 22537016

[B70] Zheng P. Z. Wang K. K. Zhang Q. Y. Huang Q. H. Du Y. Z. Zhang Q. H. (2005). Systems analysis of transcriptome and proteome in retinoic acid/arsenic trioxide-induced cell differentiation/apoptosis of promyelocytic leukemia. *Proc. Natl. Acad. Sci. U.S.A.* 102 7653–7658. 10.1073/pnas.0502825102 15894607 PMC1140456

[B71] Zhu Y.-G. Yoshinaga M. Zhao F.-J. Rosen B. P. (2014). Earth abides arsenic biotransformations. *Annu. Rev. Earth Planet. Sci.* 42 443–467. 10.1146/annurev-earth-060313-054942 26778863 PMC4712701

