# Supplementary material for: Transcriptomic Analysis Reveals Adaptive Responses of an Enterobacteriaceae Strain LSJC7 to Arsenic Exposure
Source: Front Microbiol. 2016 May 2;7:636. doi: 10.3389/fmicb.2016.00636 (PMC4852401; doi:10.3389/fmicb.2016.00636)
Supplement: Supplementary file 1 [file Data_Sheet_1.DOCX]

**Table S1.** Functional groups of differential expression genes with biologically relevant effects on LSJC7 cellular response to arsenic.

| Locus ID | Gene name | Gene annotation | As vs. control | |
| --- | --- | --- | --- | --- |
|  |  |  | Fold change | log_2_ Fold change |
|  | Arsenic network | |  |  |
| LSJC7GL000687 | *phoH* | PHOH_Protein PhoH | -2.4 | -1.3 |
| LSJC7GL004034 | *pstS* | PSTS_Phosphate-binding protein PstS | -2.1 | -1.0 |
| LSJC7GL001421 | *arsR* | ARSR2_Arsenical resistance operon repressor | 4.6 | 2.2 |
| LSJC7GL001422 | *arsD* | ARSD2_Arsenical resistance operon trans-acting repressor ArsD | 56.4 | 5.8 |
| LSJC7GL001423 | *arsA* | ARSA2_Arsenical pump-driving ATPase | 27.7 | 4.8 |
| LSJC7GL001424 | *arsB* | ARSB2_Arsenical pump membrane protein | 13.1 | 3.7 |
| LSJC7GL001425 | *arsC* | ARSC_Arsenate reductase | 13.8 | 3.8 |
| LSJC7GL002469 | *arsC* | ARSC_Arsenate reductase | 17.6 | 4.1 |
| LSJC7GL002470 | *arsB* | ARSB2_Arsenical pump membrane protein | 15.5 | 4.0 |
| LSJC7GL002471 | *arsR* | ARSR_Arsenical resistance operon repressor | 24.2 | 4.6 |
|  | NO regulation, nitrate respiration and TCA cycle | |  |  |
| LSJC7GL002585 | *nsrR* | NSRR_HTH-type transcriptional repressor NsrR | 2.4 | 1.3 |
| LSJC7GL000989 | *nrfA* | NRFA_Cytochrome c-552 | -2.3 | -1.2 |
| LSJC7GL002276 | *hmp* | HMP_Flavohemoprotein | 13.3 | 3.7 |
| LSJC7GL000437 | *sdhB* | DHSB_Succinate dehydrogenase iron-sulfur subunit | -2.4 | -1.3 |
| LSJC7GL002562 | *frdA* | FRDA_Fumarate reductase flavoprotein subunit | -2.1 | -1.1 |
| LSJC7GL001002 | *narL* | NARL_Nitrate/nitrite response regulator protein NarL | 24.4 | 4.6 |
| LSJC7GL001003 | *narX* | NARX_Nitrate/nitrite sensor protein NarX | 30.1 | 4.9 |
| LSJC7GL001004 | *narK* | NARK_Nitrite extrusion protein 1 | 138.5 | 7.1 |
| LSJC7GL001005 | *narG* | NARG_Respiratory nitrate reductase 1 alpha chain | 126.6 | 7.0 |
| LSJC7GL001006 | *narH* | NARH_Respiratory nitrate reductase 1 beta chain | 114.7 | 6.8 |

Table S1 to be continued

| Locus ID | Gene name | Gene annotation | As vs. control | |
| --- | --- | --- | --- | --- |
|  |  |  | Fold change | log_2_ Fold change |
|  | NO regulation, nitrate respiration and TCA cycle | |  |  |
| LSJC7GL001007 | *narJ* | NARJ_Nitrate reductase molybdenum cofactor assembly chaperone NarJ | 81.0 | 6.3 |
| LSJC7GL001008 | *narI* | NARI_Respiratory nitrate reductase 1 gamma chain | 44.7 | 5.5 |
| LSJC7GL002178 | *napC* | NAPC_Cytochrome c-type protein NapC | 5.2 | 2.4 |
| LSJC7GL002179 | *napB* | NAPB_Periplasmic nitrate reductase, electron transfer subunit | 8.1 | 3.0 |
| LSJC7GL002180 | *napH* | NAPH_Ferredoxin-type protein NapH | 6.8 | 2.8 |
| LSJC7GL002181 | *napG* | NAPG_Ferredoxin-type protein NapG | 6.6 | 2.7 |
| LSJC7GL002182 | *napA* | NAPA_Periplasmic nitrate reductase | 7.9 | 3.0 |
| LSJC7GL002183 | *napD* | NAPD_Protein NapD | 4.5 | 2.2 |
| LSJC7GL002184 | *napF* | NAPF_Ferredoxin-type protein NapF | 2.7 | 1.4 |
| LSJC7GL003054 | *nirB* | NIRB_Nitrite reductase [NAD(P)H] large subunit | 2.3 | 1.2 |
| LSJC7GL001279 | *narZ* | NARZ_Respiratory nitrate reductase 2 alpha chain | -3.7 | -1.9 |
| LSJC7GL001278 | *narY* | NARY_Respiratory nitrate reductase 2 beta chain | -4.4 | -2.1 |
|  | Oxidative stress response | |  |  |
| LSJC7GL002479 | *soxS* | SOXS_Regulatory protein SoxS | 5.6 | 2.5 |
| LSJC7GL002480 | *soxR* | SOXR_Redox-sensitive transcriptional activator SoxR | 4.4 | 2.2 |
| LSJC7GL000578 | *trxB* | TRXB_Thioredoxin reductase | 3.4 | 1.8 |
| LSJC7GL002377 | *trxC* | THIO2_Thioredoxin-2 | 11.4 | 3.5 |
| LSJC7GL004368 | *trxA* | THIO_Thioredoxin-1 | 2.8 | 1.5 |
| LSJC7GL003668 | *nrdH* | NRDH_Glutaredoxin-like protein NrdH | 21.3 | 4.4 |
| LSJC7GL002501 | *cpo* | PRXC_Non-heme chloroperoxidase | -3.1 | -1.6 |
| LSJC7GL004249 | *cpo* | PRXC_Non-heme chloroperoxidase | -3.1 | -1.6 |

Table S1 to be continued

| Locus ID | Gene name | Gene annotation | As vs. control | |
| --- | --- | --- | --- | --- |
|  |  |  | Fold change | log_2_ Fold change |
|  | Oxidative stress response | |  |  |
| LSJC7GL000272 | *osmC* | OSMC_Peroxiredoxin OsmC | -2.8 | -1.5 |
| LSJC7GL001449 | *katG* | KATG_Catalase-peroxidase | -2.9 | -1.5 |
| LSJC7GL001696 | *katE* | CATE_Catalase HPII | -2.1 | -1.1 |
| LSJC7GL000563 | *hcr* | HCR_NADH oxidoreductase hcr | 5.6 | 2.5 |
| LSJC7GL000564 | *hcp* | HCP_Hydroxylamine reductase | 10.2 | 3.3 |
| LSJC7GL001469 | *marR* | MARR_Multiple antibiotic resistance protein MarR | 13.2 | 3.7 |
| LSJC7GL001470 | *marA* | MARA_Multiple antibiotic resistance protein MarA | 4.9 | 2.3 |
| LSJC7GL001471 | *marB* | MARB_Multiple antibiotic resistance protein MarB | 2.2 | 1.0 |
|  | Iron uptake and iron-sulfur cluster regulation | |  |  |
| LSJC7GL002604 | *ytfE* | YTFE_Iron-sulfur cluster repair protein YtfE | 72.2 | 6.2 |
| LSJC7GL002922 | *erpA* | ERPA_Iron-sulfur cluster insertion protein ErpA | 6.0 | 2.6 |
| LSJC7GL003115 | *nfuA* | NFUA_Fe/S biogenesis protein NfuA | 8.2 | 3.0 |
| LSJC7GL003013 | *bfd* | BFD_Bacterioferritin-associated ferredoxin | 22.8 | 4.5 |
| LSJC7GL000343 | *fepD* | FEPD_Ferric enterobactin transport system permease protein FepD | 3.3 | 1.7 |
| LSJC7GL003110 | *feoA* | FEOA_Ferrous iron transport protein A | 5.5 | 2.4 |
| LSJC7GL002746 | *fhuF* | FHUF_Ferric iron reductase protein FhuF | 7.7 | 2.9 |
| LSJC7GL002916 | *fhuA* | FHUA_Ferrichrome-iron receptor | 3.0 | 1.6 |
| LSJC7GL003918 | *exbB* | EXBB_Biopolymer transport protein ExbB | 5.3 | 2.4 |
| LSJC7GL002263 | *iscR* | ISCR_HTH-type transcriptional regulator IscR | 3.6 | 1.8 |
| LSJC7GL003012 | *bfr* | BFR_Bacterioferritin | -2.4 | -1.3 |

Table S1 to be continued

| Locus ID | Gene name | Gene annotation | As vs. control | |
| --- | --- | --- | --- | --- |
|  |  |  | Fold change | log_2_ Fold change |
|  | DNA repair | |  |  |
| LSJC7GL001569 | *smrA* | SMRA_Probable DNA endonuclease SmrA | 2.1 | 1.1 |
|  | Proteases | |  |  |
| LSJC7GL004162 | *hslV* | HSLV_ATP-dependent protease subunit HslV | 5.6 | 2.5 |
| LSJC7GL004161 | *hslU* | HSLU_ATP-dependent protease ATPase subunit HslU | 2.2 | 1.1 |
| LSJC7GL003014 | *clpP3* | CLPP3_ATP-dependent Clp protease proteolytic subunit 3 | 2.3 | 1.2 |
| LSJC7GL001755 | *clpP3* | CLPP3_ATP-dependent Clp protease proteolytic subunit 3 | -3.2 | -1.7 |
| LSJC7GL004299 | *aprE* | APRE_Alkaline protease secretion protein AprE | -2.3 | -1.2 |
| LSJC7GL001127 | *NGR_a01040* | Y4WA_Uncharacterized zinc protease y4wA | -2.2 | -1.1 |
|  | Protein metabolism and chaperone | |  |  |
| LSJC7GL003744 | *rpsI* | RS9_30S ribosomal protein S9 | 4.2 | 2.1 |
| LSJC7GL003017 | *rpsG* | RS7_30S ribosomal protein S7 | 2.8 | 1.5 |
| LSJC7GL002597 | *rpsF* | RS6_30S ribosomal protein S6 | 2.6 | 1.4 |
| LSJC7GL002987 | *rpsD* | RS4_30S ribosomal protein S4 | 2.1 | 1.1 |
| LSJC7GL003004 | *rpsC* | RS3_30S ribosomal protein S3 | 3.5 | 1.8 |
| LSJC7GL003884 | *rpsU* | RS21_30S ribosomal protein S21 | 3.4 | 1.8 |
| LSJC7GL002794 | *rpsT* | RS20_30S ribosomal protein S20 | 3.9 | 2.0 |
| LSJC7GL002935 | *rpsB* | RS2_30S ribosomal protein S2 | 2.7 | 1.5 |
| Novel00020 | *rpsS* | RS19_30S ribosomal protein S19 | 5.0 | 2.3 |
| LSJC7GL002599 | *rpsR* | RS18_30S ribosomal protein S18 | 2.6 | 1.4 |
| LSJC7GL003001 | *rpsQ* | RS17_30S ribosomal protein S17 | 9.0 | 3.2 |
| Novel00023 | *rpsQ* | RS17_30S ribosomal protein S17 | 7.8 | 3.0 |

Table S1 to be continued

| Locus ID | Gene name | Gene annotation | As vs. control | |
| --- | --- | --- | --- | --- |
|  |  |  | Fold change | log_2_ Fold change |
|  | Protein metabolism and chaperone | |  |  |
| LSJC7GL003795 | *rpsO* | RS15_30S ribosomal protein S15 | 2.6 | 1.4 |
| LSJC7GL003018 | *rpsL* | RS12_30S ribosomal protein S12 | 2.7 | 1.4 |
| LSJC7GL000599 | *rpsA* | RS1_30S ribosomal protein S1 | 2.3 | 1.2 |
| LSJC7GL002600 | *rplI* | RL9_50S ribosomal protein L9 | 3.6 | 1.9 |
| LSJC7GL002393 | *rplL* | RL7_50S ribosomal protein L7/L12 | 2.4 | 1.3 |
| LSJC7GL003009 | *rplD* | RL4_50S ribosomal protein L4 | 2.6 | 1.4 |
| LSJC7GL004111 | *rpmG* | RL33_50S ribosomal protein L33 | 4.7 | 2.2 |
| LSJC7GL000726 | *rpmF* | RL32_50S ribosomal protein L32 | 2.5 | 1.3 |
| LSJC7GL003010 | *rplC* | RL3_50S ribosomal protein L3 | 2.4 | 1.3 |
| LSJC7GL003002 | *rpmC* | RL29_50S ribosomal protein L29 | 8.6 | 3.1 |
| LSJC7GL004110 | *rpmB* | RL28_50S ribosomal protein L28 | 4.0 | 2.0 |
| LSJC7GL001971 | *rplY* | RL25_50S ribosomal protein L25 | 3.6 | 1.8 |
| LSJC7GL002999 | *rplX* | RL24_50S ribosomal protein L24 | 2.1 | 1.1 |
| LSJC7GL003008 | *rplW* | RL23_50S ribosomal protein L23 | 2.3 | 1.2 |
| LSJC7GL003005 | *rplV* | RL22_50S ribosomal protein L22 | 3.3 | 1.7 |
| LSJC7GL003007 | *rplB* | RL2_50S ribosomal protein L2 | 2.6 | 1.4 |
| LSJC7GL003694 | *rplS* | RL19_50S ribosomal protein L19 | 2.2 | 1.1 |
| LSJC7GL003003 | *rplP* | RL16_50S ribosomal protein L16 | 4.5 | 2.2 |
| LSJC7GL003000 | *rplN* | RL14_50S ribosomal protein L14 | 2.1 | 1.0 |
| LSJC7GL002392 | *rplJ* | RL10_50S ribosomal protein L10 | 2.0 | 1.0 |
| LSJC7GL003706 | *clpB* | CLPB_Chaperone protein ClpB | 2.1 | 1.1 |

Table S1 to be continued

| Locus ID | Gene name | Gene annotation | As vs. control | |
| --- | --- | --- | --- | --- |
|  |  |  | Fold change | log_2_ Fold change |
|  | Protein metabolism and chaperone | |  |  |
| LSJC7GL002790 | *dnaK* | DNAK_Chaperone protein DnaK | 8.4 | 3.1 |
| LSJC7GL002791 | *dnaJ* | DNAJ_Chaperone protein DnaJ | 4.1 | 2.0 |
| LSJC7GL000180 | *htpG* | HTPG_Chaperone protein HtpG | 4.8 | 2.3 |
| LSJC7GL003077 | *hslO* | HSLO_33 kDa chaperonin | 3.2 | 1.7 |
| LSJC7GL002551 | *groL* | CH60_60 kDa chaperonin | 3.0 | 1.6 |
| LSJC7GL002550 | *groS* | CH10_10 kDa chaperonin | 5.7 | 2.5 |
| LSJC7GL000251 | *ppiB* | PPIB_Peptidyl-prolyl cis-trans isomerase B | 2.4 | 1.3 |
| LSJC7GL002602 | *fklB* | FKBB_FKBP-type 22 kDa peptidyl-prolyl cis-trans isomerase | 3.1 | 1.6 |
| LSJC7GL003023 | *fkpA* | FKBA_FKBP-type peptidyl-prolyl cis-trans isomerase FkpA | 3.3 | 1.7 |
| LSJC7GL003015 | *tufA* | EFTU_Elongation factor Tu | 3.0 | 1.6 |
| LSJC7GL003016 | *fusA* | EFG_Elongation factor G | 2.8 | 1.5 |
| LSJC7GL002387 | *tufA* | EFTU_Elongation factor Tu | 2.9 | 1.6 |
| LSJC7GL002937 | *tsf* | EFTS_Elongation factor Ts | 5.1 | 2.4 |
| LSJC7GL000133 | *tig* | TIG_Trigger factor | 2.3 | 1.2 |
| LSJC7GL004141 | *cpxP* | CPXP_Periplasmic protein CpxP | 9.9 | 3.3 |
| LSJC7GL000649 | *hspQ* | HSPQ_Heat shock protein HspQ | 2.4 | 1.2 |
| LSJC7GL000368 | *cspE* | CSPE_Cold shock-like protein CspE | 2.2 | 1.1 |
| LSJC7GL000571 | *cspD* | CSPD_Cold shock-like protein CspD | 2.3 | 1.2 |
| LSJC7GL004067 | *ibpA* | IBPA_Small heat shock protein IbpA | 12.2 | 3.6 |
| LSJC7GL004068 | *ibpB* | IBPB_Small heat shock protein IbpB | 3.3 | 1.7 |

**Table S2. Summary of RNA-seq dataset.**

| Sample | Total reads | Total mapped | Reads mapped | Clean bases |
| --- | --- | --- | --- | --- |
| Control | 19,693,478 | 19,341,008 | 98.21% | 1.96G |
| As | 24,546,836 | 24,406,576 | 99.43% | 2.46G |

**Table S3. The information of the differentially expressed genes (Excel file separately attached)**

**Table S4. Primers used in RT-qPCR**

| Gene | Forward (5’-3’) | Reverse (5’-3’) |
| --- | --- | --- |
| 16S | GTCTTGACATCCACAGAACC | CCATTGTAGCACGTGTGTAG |
| *hmp* | CACTATCTGCCAGGTCAGTA | GTGGCAAGCTGCACAATATCA |
| *trxB* | AGCGCTGTATCTGTCCAACA | TCGCCGGTCACTTCATCAA |
| *nfuA* | TGACGACGCACCGCTGATTGA | GTCCACCATAGAGCAACCGTTA |
| *napB* | AATACCAACCGCTGCCTGGCA | ACGGCTCGAAGGTGTTATCAAC |
| *napA* | TGTGCTGGAGCACTGGCATA | CCACAGTGGTAATAACTTCGC |
| *soxS* | GGATGTTCCGCACCGTGAA | GTCTGCTGCGACACATAGC |
| *soxR* | CGATATACGCGTGATGTGCTG | CGACAGCTGCTTCCACTCT |
| *erpA* | TCTGATTGCAGATGAAGAG | AATGCTAAATGACGAGCCAC |
| *arsC* | GTGAAACTCATTGCTGATATG | TTATCAGCAGCGGATACTGC |
| *narK* | GTGATGAGCAGGCAATGCGTG | TCAGGTCAAGCGACGTGCCGA |
| *arsD* | CAACCTATGAGCTTCGTTCAG | TACTTTCTCCAGCGGAATGCC |
| *nrfA* | GTATCCATATGCATGCGCCTGA | GGAGATATCCGGTAGTCGAAC |
| *phoH* | TCCAGGCCGATGAAGATCTC | CGGGCGTAAGCAGTACTGCATA |
| *pstS* | CTGCCGACGGCAAGTCAGTCA | CAGAATGAAGGTAGTGGAGGTG |
| *katE* | CCTGCGGTGATATTGCCTCCA | GTAGTGCTGTCTGCGCTCAGT |
| *bfr* | AGGATCTCGGCAAGCTGCGCA | ATGAACGCTGTCAGCATACGC |
| *narZ* | ACCTGCTGATGCTCACCATGT | TTGCGGATGATGATGAACTC |
| *narG* | ACGACTGGATTGAAGTGTTC | ATACGGGTCACGGAGTTATGA |
