# Supplementary figures and images for: Transcriptomic Analysis Reveals Adaptive Responses of an Enterobacteriaceae Strain LSJC7 to Arsenic Exposure
Source: Front Microbiol. 2016 May 2;7:636. doi: 10.3389/fmicb.2016.00636 (PMC4852401; doi:10.3389/fmicb.2016.00636)

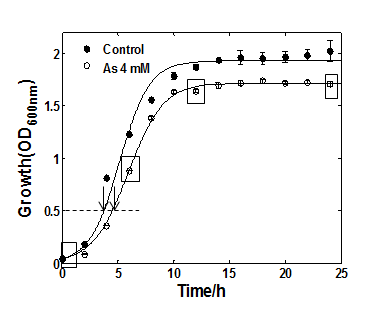

Supplement: FIGURE S1 — Growth rates of LSJC7 under 4 mM As(V) stress. The arrows represent the sampling time for transcriptome, and the boxes represent the sampling time for As species analysis. [file Image_1.TIF]
